# Birth Defects Registries in the Genomics Era: Challenges and Opportunities for Developing Countries

**DOI:** 10.3389/fped.2014.00060

**Published:** 2014-06-16

**Authors:** Meow-Keong Thong

**Affiliations:** ^1^Department of Paediatrics, Faculty of Medicine, University of Malaya, Kuala Lumpur, Malaysia

**Keywords:** congenital anomalies, birth defects, developing countries, birth defect register

## Abstract

Birth defects or congenital anomalies are one of the major causes of disability in developed and developing countries. Data on birth defects from population-based studies originating from developing countries are lacking. Increasingly, there is a shift to genetic testing and genomics study of birth defects. However, the translation from bench findings to bedside medicine has been muted. There is a need to address this imbalance where congenital anomalies remained the top etiology for neonatal mortality in developing countries. To build capacity in low resource countries, there is a need for accurate collection and ascertainment of birth defects in developing countries. The systematic collection and analysis of data on major birth defects using birth defects registries (BDRs) are an integral part of all clinical genetic services. Healthcare planners in developing countries must be aware of the advantages and limitations of BDRs. Despite the advent of the genomics era, BDRs are essential to the planning and developing care and prevention services at local and national levels, particularly in low resource or developing countries.

## Introduction

Birth defects or congenital anomalies are one of the major causes of disability in developed and developing countries (1, 2). Data on birth defects from population-based studies originating from developing countries in the Asia-Pacific region are lacking (3). The March of Dimes estimated 7.4 million infants are born each year with a serious birth defect. Of these births, 94% occur in middle and low income countries (4). Over 3.3 million children under age five die annually from birth defects. The etiology for birth defects is heterogenous and there are a number of preventative strategies that can be adopted to reduce them (5, 6). With the development of newer technologies in genomics medicine, it is possible to characterize the molecular bases of many rare disorders and birth defects (7, 8). Increasingly, there are databases established for a single specified condition to study the phenotype–genotype correlations or genomic variations within the disorder (9). Hence, there is a need to re-visit the roles of birth defects registers. This is especially important in parts of the world where congenital anomalies remained a major cause of neonatal mortality and morbidity (10).

The systematic collection and analysis of data on major birth defects using a birth defect register (BDR) has traditionally being an integral part of a clinical genetic service (11, 12). This requires the strengthening of medical genetic services in low- and middle-income countries (13) Reliable data birth defects rely on the on-going surveillance on the types, birth prevalence, severity, and outcome of children with birth defects. Sources of information that contribute to the BDR include health professionals, special treatment centers, and private hospitals and healthcare practices, autopsy services, and laboratory diagnostic services. The notification of birth defects may be done under a voluntary basis or under the authority of legislation. The methods of ascertainment used are important as active system of case notification will yield a higher number of cases than passive notification. There is a need to obtain ethical approval to review patient case files from all hospitals and healthcare facilities to ensure that patient privacy is protected and confidentiality of information assured.

## Functions and Limitations of a Birth Defects Register

The functions of a BDR are manifold. It helps to establish local prevalence rates for birth defects in the local population as well as to facilitate and determine accurate baseline incidence and detection of trends of birth defects. This will be essential to the planning of health care facilities, allocation of appropriate health resources required for the design, and implementation of various preventative programs, training of medical, and paramedical staff to provide the skills to recognize major birth defects within the framework of public health (11) and facilitating access to genetic counseling services (14). Genetic counseling is not easily available in most health services and a BDR may serve as a nucleus to train providers of genetic counseling, population screening, and public education (14, 15). With such amenities, pre-natal diagnosis for specific genetic disease can be achieved for at-risk families A BDR also function to monitor the occurrence of defects over time and by geographical area to allow earlier detection of the emergence of any new teratogen and further investigations of suspected teratogens (16, 17). It can be used to perform epidemiological studies to identify the causation of birth defect, for clinical research (18), and to increase community knowledge about birth defects through education. Empowerment of the members of the public with knowledge regarding birth defects is essential to ensure success of any preventive programs. For example, valproate should not be first-line anti-epileptic drugs (AED) for women who are considering pregnancy. In this situation, this drug is best avoided if other effective but safer AEDs can be found for each individual woman’s seizure disorder (19). When valproate cannot be avoided in pregnancy, the lowest possible effective dose should be prescribed in two to three divided doses, preferably as monotherapy. Women exposed to valproate in pregnancy should be given periconceptional folic acid and followed up in a high risk pregnancy clinic (20).

It is to be expected that there are a number of difficulties and limitations arising from the planning and maintenance of a BDR. Many medical practitioners are bewildered by the various terms used in a BDR. For example, the terms “congenital abnormalities,” “congenital disorders,” “congenital anomalies or malformations,” and “birth defects” have been used interchangeably in some centers although specific definitions are available for the above. The March of Dimes used “birth defects” and these include abnormalities in structure or function, including metabolism, which are present from birth. The World Health Organization (WHO) Human Genetics Programme (HGN) prefers “congenital disorder,” which is defined as any potential pathological condition arising before birth, including all disorders caused by environmental, genetic, or unknown factors, whether they are evident at birth or become manifest later in life. The WHO Burden of Disease Unit uses “congenital anomalies” defined as macroscopic morphological anomalies present from birth. This excludes functional birth defects, disability, common single gene disorders such as cystic fibrosis and glucose-6-phosphate dehydrogenase deficiency and inborn errors of metabolism. In a report of a joint WHO-March of Dimes meeting in 2006, it was decided that the term “birth defect” is synonymous with the term “congenital disorder” as defined and used by the HGN (13).

Another difficult issue has been ascertainment of infants with multiple birth defects. This may require the assessment of a clinical geneticist to make a syndromic diagnosis or to perform the appropriate investigations. Increasingly, pre-natal diagnosis and termination of pregnancies are performed in many communities. This may reduce the births of newborns with birth defects but the true prevalence remains the same. Hence, many BDR include termination of pregnancies in the data collection. The timing of the case ascertainment may also cause variations in the data collection. Increasingly, there are many databases established to register patients with some specific disorders and the main objective is to study the phenotype–genotype correlations or genomic variations within the disorder (9). These databases may draw away further financial support and genetic expertise away from the BDR.

There have been few studies in developing countries on the cost-effectiveness of BDR and long-term benefits are uncertain (11). Many medical or health care planners are reluctant or unable to commit public funds for medical registers due to the lack of definitive evidence of cost-effectiveness. There may be underutilization of the data in the BDR due to lack of expertise or research funding, for example, to maintain a teratogen warning system. There is a need to increase public and professional awareness on birth defects and to facilitate research collaboration, locally, and regionally. Collaboration with the public health sector or “public health genomics” may result in specific targeted preventive programs for birth defects. With increased public or consumer sensitization to birth defects, the issue of stigmatization and marginalization of individuals with birth defects may be further reduced (14). Hence, it is vital for healthcare planners to be aware of these challenges and opportunities provided by a BDR.

## Setting Up a Birth Defects Register

The setting up of a Birth Defects Register requires careful and detailed planning with extensive consultation with various experts and open collaboration with many professional groups and healthcare services. The staff of the BDR may consists of clinical geneticist, pediatrician, obstetrician, representative from the maternal and child health service, epidemiologist and biostatistician, data manager, nursing staff, and clerical assistants. It also requires assured sources of funding as the health returns from a BDR may not be evident for many years. Frequently, experts from the public health and community genetics work closely together to design a program that will be the most suitable for the local situation. A BDR may be population-based or hospital-based.

A BDR may need to decide whether to include all major birth defects or just sentinel birth defects. Ideally, all major birth defects should be included in a BDR. Some BDR have advocated sentinel defects only and these are usually birth defects that are evident at birth on inspection. The disadvantage of this is that major birth defects arising in certain population or following a teratogenic effect may be missed if reporting is based on sentinel findings that are not involved. Due to logistical difficulties, some BDR may opt to report birth defects in certain regions or localities only, rather than cover the entire population. Another important aspect is that the BDR must operate within the framework of the current health system and services. A BDR is usually governmental in origin although non-governmental organizations may be an active participant in the program. An important factor for a BDR to consider is to provide timely and up-to-date feedback to stakeholders of the program. This may include the members of the medical profession, health care planners, and hospitals and services that contributed to the BDR.

## Birth Defects Registries in the Asia-Pacific Region

In South-East Asia, only Singapore has a National BDR, which was established in 1992. It is population-based and funded by the government. It collects all major and minor birth defects but not inborn errors of metabolism. The common birth defects are congenital heart defects, cleft lip and palate, gastrointestinal defects, neural tube defects, and limb defects (21). More than 20% of medically certified infant deaths in Malaysia were classified as due to birth defects in 1990. As the data collected were hospital-based, a pilot study to set up a population-based BDR was initiated in 2000 (10). The results showed the prevalence of birth defects were 1 in 70 and several risk factors were identified. It recommended pre-natal and antenatal screening for insulin dependent diabetes, genetic counseling, and investigating causes for previous abortions and public education on avoidance of teratogens. It also emphasized the role of periconceptional folic acid supplementation or food fortification. Of concern was that only 15% of the birth defects were detected during the antenatal period (22). The main birth defects were chromosomal disorders, congenital heart disease, cleft lip and palate, club feet, and central nervous system defects. The population-based study was the first such study in Malaysia. Several hospital-based birth defects registers were also started (23). As a long-term follow-up, a section on birth defects were included in the Malaysian National Neonatal Registry (MNNR), beginning from 2005. This has yielded important data on many birth defects, for example, the MNNR provided detailed information on the incidence of neural tube defects in various localities in Malaysia, identified needs and proposed various targeted recommendations (24). In 2006, the Ministry of Health of Malaysia reported that the number one cause of under-five deaths were congenital anomalies (25). In the Philippines, several pilot projects on congenital anomalies were started (26). In 2008, a Birth Defects Surveillance Project was announced and was coordinated from the Institute of Human Genetics, National Institutes of Health with funding provided by the Department of Health, University of Philippines and March of Dimes Foundation. It was both hospital-based and community based and only physical defects were included. The major birth defects found in the pilot studies were ankyloglossia, multiple birth defects (not elsewhere classified), cleft lip and palate, talipes equinovarus, and anencephaly. The Sixth International Conference on Birth Defects and Disabilities in the Developing World was recently held in Cebu City, Philippines on the 10–13th of November 2013.

## Conclusion

The systematic collection and analysis of data on major birth defects using BDRs are an integral part of all clinical genetic services. Healthcare planners in developing countries must be aware of the advantages and limitations of BDRs. Despite the advent of the genomics era, BDRs are essential to the planning and developing care and prevention services at local and national levels, particularly in low resource or developing countries.

## Conflict of Interest Statement

The author declares that the research was conducted in the absence of any commercial or financial relationships that could be construed as a potential conflict of interest.
